# Comment on: Prevalence of transfusion-transmitted infections in Saudi Arabia blood donors: A nationwide, cross-sectional study

**DOI:** 10.15537/smj.2023.4.20230104

**Published:** 2023-04

**Authors:** Nour Al-Mozain, Maha A. Badawi, Mohammad A. Alfhili

**Affiliations:** Department of Pathology and Laboratory Medicine, King Faisal Specialist Hospital and Research Centre, Riyadh, Kingdom of Saudi Arabia.; Department of Hematology, Faculty of Medicine, King Abdulaziz University, Jeddah, Kingdom of Saudi Arabia.; Chair of Medical and Molecular Genetics Research, Department of Clinical Laboratory Sciences, College of Applied Medical Sciences, King Saud University, Riyadh, Kingdom of Saudi Arabia

*To the Editor*

We have read with interest the article published in Saudi Medical Journal on Prevalence of transfusion-transmitted infections in Saudi Arabia blood donors nationwide; cross-sectional study.^1^

Safe blood supply has always been considered fundamental to the modern healthcare system in Saudi Arabia. Despite the global call for patient blood management implementation and recent innovations in pharmaceutical alternatives, the demand for blood transfusion remains relatively constant globally and in Saudi Arabia. This is the result of the rapid growth in healthcare in terms of the ability to medically or surgically treat advanced conditions that were previously deemed incurable. Blood transfusion or (hemotherapy) is essential for many of these therapeutic interventions. There are no published statistics about blood utilization nationally. Compared to published numbers from other countries, as in the UK,^2^ we assume that the estimated blood utilization is around 1.5 million units annually based on the size of our population. Humans remain the only source of blood and blood products despite the ongoing trials of having artificial blood. Safety assurance of the collected blood against “known and yet-to-be-known” transfusion-transmitted infections (TTI) remains a major challenge in the field. We appreciate the efforts of the Ministry of Health (MOH) team in publishing valuable statistics about TTI detection rates in the Kingdom, which national professionals in blood banking and transfusion medicine were eager to know.^1^ The data shared1 show the detected rates of TTI are generally acceptable, but we found the rate of positive testing for hepatitis B virus nucleic acid testing (NAT) of 2.5% in Riyadh alarming. High rates of TTI detection among potential donors indicate the need for improved donor selection. We identify 2 areas for improvement in the donor selection process on a national level. The first is related to the practice of accepting family replacement donors. It is well recognized that non-voluntary donation affects the credibility and transparency of the donor, which may allow high-risk donors to enter the donor pool easily. This increases both the rates of TTIs detected by screening tests and the chances of transfusion-transmitted infections from a donor in the window period. The second area for improvement is the adoption of the Blood Donor History Questionnaire (DHQ) developed by Western organizations. Internationally developed DHQs are created to screen for well-recognized risk factors in the country of origin. We advocate for modification of DHQ to address relevant local risk factors, such as undocumented marriages. Moreover, a modified DHQ will guide the Saudi blood banking community in donor screening as many countries move towards individualized risk assessment regardless of the donor’s gender or sexual orientation. We also hope that a modified DHQ would address TTI that we do not currently screen for, including West Nile Virus, Chikungunya Virus, Hepatitis E, and Dengue Fever. As the world becomes a small village, guidance is needed even for infections not endemic in the Kingdom. We wonder if testing may be needed for some of these infections or whether a travel-based deferral may be adequate.

Suggested policy and practice changes require more national data on the incidence of TTIs, trends, and risk factors. Acquiring and sharing these data will further increase trust among prescribers and recipients of blood. The residual risk of TTIs in the Kingdom is expected to be reassuring, given the advanced technologies used for donor screening. However, estimating that risk requires access to data about repeat donors who are positive for NAT only (NAT yield).^3^ This emphasizes the point raised by the authors regarding the need to more detailed information when discussing TTIs in the Kingdom.^1^

In addition to the anticipation of more data sharing by MOH, we also encourage all those working in the fields of transfusion medicine, public health, and infectious diseases in Saudi Arabia to conduct and publish about TTI. Single cases of transmission of an infection may lead to significant findings and raise the need for major changes. In 2007, researchers from Germany discovered that the sensitivity of several NAT assays for HCV did not perform as expectedly with HCV genotype 2b while investigating a case of HCV transmission in the window period.^4^ Do the commercially available NAT assays have the same performance levels for genotype 4, the most prevalent genotype in the Saudi population^5^ as they do with genotype 1, the most common genotype globally? Only research and surveillance can tell.

As the authors suggested,^1^ a national blood supplier will be a great step forward towards collection of complete data in a standardized manner. Funding organizations have a duty to make TTI research as a priority when evaluating potential projects for grants, and research support opportunities will be needed to allow more complex research and data availability. Protecting the blood supply is a joint effort, and we believe that the road to the best policies starts with availability of accurate and detailed data.

*Reply from the Author*

We thank Almozain and Badawi for their insightful comments. We agree that the current rate of positive hepatitis B virus nucleic acid testing among blood donors in the central region is alarming and urges the implementation of compatible blood donation strategies. The 2 areas for improvement suggested by Almozain and Badawi are of great importance to decrease the rates of transfusion-transmitted infections detected by screening tests. In our recently published work,^6^ we discussed the necessity of establishing a centralized blood bank management system on a national level to avoid the several shortcomings observed with the current fragmented system and hope the Health Sector Transformation Program will take these comments into consideration.
